# Urinary Arsenic Concentration and Its Relationship with Bronchial Asthma in Children from Arica, Chile

**DOI:** 10.3390/toxics10100625

**Published:** 2022-10-19

**Authors:** María Pía Muñoz, Verónica Iglesias, Marta Saavedra, Gina Saavedra, Karla Yohannessen, Paulina Pino

**Affiliations:** 1 Programa Doctorado en Salud Pública, Escuela de Salud Pública, Facultad de Medicina, Universidad de Chile, Independencia 939, Santiago 8380453, Chile; 2 Programa de Epidemiología, Escuela de Salud Pública, Facultad de Medicina, Universidad de Chile, Independencia 939, Santiago 8380453, Chile; 3 Departamento de Salud Pública, Secretaría Regional Ministerial de Salud Arica y Parinacota, Maipú 410, Arica 1000426, Chile

**Keywords:** bronchial asthma, child, environmental exposure, ethnicity, population surveillance, urinary arsenic

## Abstract

In the city of Arica, northern Chile, the population has been involuntarily exposed to arsenic of natural and anthropogenic origin. This study aims to evaluate the association between urinary arsenic concentration and bronchial asthma diagnosis in the children of Arica. A cross-sectional analysis of a database of 1892 subjects under 18 years of age enrolled in the Environmental Health Centre between 2009 and 2021 was carried out. Arsenic exposure was obtained from a urine sample and bronchial asthma diagnosis from the database of the system for the management of explicit health guarantees. Logistic regression models were used to assess the association between inorganic arsenic and asthma. The median inorganic arsenic was 15 μg/L, and the prevalence of asthma was 7.4%. After adjusting for sex, age, ethnicity, and urinary creatinine, children with the highest tertile of urinary arsenic concentration (≥21.4 μg/L) had a greater chance of developing asthma (odds ratio (OR) 1.90; 95% confidence interval (CI) [1.13–3.18]). When exploring the modifying effect of ethnicity, the association increased among children belonging to any ethnic group (OR 3.51, 95%CI [1.43–8.65]). These findings suggest a relationship between arsenic exposure and bronchial asthma in children. While further studies are needed to assess the impact of arsenic on respiratory health, mitigation efforts to reduce arsenic exposure should be maintained.

## 1. Introduction

Asthma is a common childhood disease, characterized by inflammation and airway obstruction. Its long-term impacts on lung function and quality of life in children are a matter of concern [1]. Worldwide, the average prevalence of asthma is 10.8% in children aged 6 to 7 years and 13.8% in children aged 13 to 14 years [2], while these figures are 10.7% and 14.9%, respectively, in Chilean schoolchildren [3]. It has been reported that the prevalence of asthma is higher in children from high-income families; however, the severity of symptoms related to asthma is higher in children of lower socio-economic level [4].

Environmental risk factors associated with asthma include allergens, air pollution, tobacco smoke, pesticide exposure, and heavy metals [5,6,7,8,9,10,11]. Several studies have evaluated the association between childhood asthma with lead and cadmium exposure [9,10,12,13,14], while the relationship with arsenic has been scarcely studied [15,16]. The susceptibility to developing arsenic-induced asthma is supported by its modulating effect on the immune system. Two studies have noted that arsenic exposure is related to asthma of allergic origin in adults, as arsenic was positively associated with serum levels of Immunoglobulin E (IgE) [17] and type 2 cytokines (Th2) [18]. 

The city of Arica is located on the Pacific Ring of Fire and, along with other Latin American cities, shares the characteristic of natural arsenic contamination of water [19]. Arica is an urban city, where water is treated to meet the national standard of 10 µg/L [20]. However, major concerns arose in the 1990s after the emergence of slums in the vicinity of abandoned toxic waste dumps with high concentrations of arsenic and other metals from trades with Sweden. In 1998, the toxic wastes were removed, and the Regional Government of Arica and Parinacota developed an intervention program in exposed areas to control exposure to metals and the associated health effects [21,22,23].

In the context of the intervention, a comprehensive health program was developed including the evaluation, monitoring, diagnosis, and treatment of the possible effects of exposure to metals, giving rise to a registry of information on the affected population. The present study aims to evaluate the association between urinary inorganic arsenic concentration and asthma in the population under 18 years registered in the Environmental Health Center of Arica from 2009 to 2021. 

## 2. Materials and Methods

### 2.1. Design and Study Population

A cross-sectional analysis was performed on a database provided by the Regional Ministerial Secretariat (acronym in Spanish, SEREMI) of Health of the Region of Arica and Parinacota, containing 13,543 records from the Environmental Health Center (EHC) between the years 2009 and 2021. In the context of Law No. 20,590 [24], the EHC collects information on the exposure and health of the population exposed to heavy metals. For this study, information of children under 18 years of age was considered. The criterion for children under 18 to access the benefits of Law No. 20,590 was to have resided for at least 6 months in one of the neighborhoods classified by the authority as a “high environmental risk area”. The cut-off date considered in the Law was 29 May 2012. For this reason, we do not include children under 8 years of age [24].

### 2.2. Measures

#### 2.2.1. Inorganic Arsenic Concentration

These secondary data were provided by the SEREMI of Health of Arica y Parinacota. The concentration of inorganic arsenic was determined from a urine sample requested during enrollment at the EHC. The analysis was performed by atomic absorption spectrophotometry with hydride generation (AAS-HG) at the Occupational Health Laboratory of the Institute of Public Health from 2009 to 2015 (96% of urine samples) and in 2016 at the Environmental and Occupational Public Health Laboratory of Arica. Both laboratories are accredited with the ISO/IEC 17025:2017, a standard that aims to certify the technical competence and reliability of analytical results. The limit of detection (LOD) was 1.99 μg/L. Concentrations below the LOD (*n* = 9) were assigned the value 0.99 μg/dL (LOD/2). Urinary creatinine concentration (gr/L) was also measured.

#### 2.2.2. Diagnosis of Asthma

Diagnosis of bronchial asthma was obtained from the database of the system for the management of explicit health guarantees (acronym in Spanish, SIGGES) and also were provided by the SEREMI of Health. Nationwide, SIGGES registers information of individuals accessing benefits associated with a given health problem in a prioritized program. For bronchial asthma, the benefits include medical diagnosis (e.g., tests) and treatment (e.g., medicines and supplies) [25]. The medical diagnostic process begins with the evaluation of the history of repeated episodes of suspected bronchial asthma, which may result in treatment or tests (e.g., baseline spirometry, chest X-ray, and bronchial provocation) performed to confirm the diagnosis, which are reported in the system [26,27]. The bronchial asthma history record was registered as a dichotomous variable (Yes/No).

#### 2.2.3. Covariates

Information on personal data including age (years), sex (female: 0, male: 1), family member with asthma (no: 0, yes: 1), and ethnicity (Aymara, Diaguitas, Atacameños, Coya, Mapuche, Afrodescendants, Quechua, Rapanui, and Yamanes) were obtained from the SIGGES database. Ethnicity was collapsed into three categories: belonging to any ethnic group, not belonging to any ethnic group, and undefined.

The variable sector of residence (outside the exposed sector: 1, other sectors of exposure (maestranza and port sector): 2, and sector F: 3 (exposed)) was obtained from the environmental health center database. The identification of the exposed areas (sector F, maestranza, and the port sector) is based on the geo-referenced results of soil samples taken by three independent institutions between 2006 and 2009 [21].

### 2.3. Data Analysis

Categorical variables are presented as frequency and percentage, and continuous variables are presented as median and interquartile range (IQR). The Mann–Whitney and Kruskal–Wallis non-parametric tests were conducted to compare the median of inorganic arsenic concentration, according to the characteristics of participants. Pearson’s Chi-square test and Fisher’s exact test were used to compare the prevalence of asthma among socio-demographic variable categories [28,29]. 

The association between urinary arsenic concentration and bronchial asthma, adjusted for confounding variables described in the literature, such as age, sex [30], and ethnicity [31,32], was assessed through multiple logistic regression models. The results are presented as odds ratios (OR) and respective 95% confidence intervals (CI). 

In addition, we explored whether ethnicity modifies the effect of arsenic on asthma. To assess effect modification, we stratified the overall analysis by ethnicity categories [33]. The statistical package STATA version 16.0 (StataCorp., College Station, TX, USA) was used for data analysis.

## 3. Results

Of the 2025 individuals under 18 years of age, 1892 (93.4%) had information on urine arsenic concentration. Those lacking urinary arsenic data (*n* = 133) were younger, with a median (IQR) age of 11 (10–13) years, and presented a significantly lower proportion of males (39.9%) than those with complete information (*p* < 0.05).

Table 1 shows the general characteristics of the population. The median (IQR) age was 14 (12–16) years, and 31.1% belonged to any ethnic group, of which 65% were Aymara. 

The median inorganic arsenic concentration was 15 μg /L (IQR: 7.0–26 μg /L). As it had a non-normal distribution (*p* < 0.001, Shapiro–Wilk test), the variable was categorized into tertiles (T) of exposure, with the following cut-off points: T1 ≤ 10 μg /L, T2 10.1–21.3 μg /L, and T3 ≥ 21.4 µg/L.

Table 2 describes the concentration of inorganic arsenic in urine and the prevalence of asthma, according to individual characteristics. The median of inorganic arsenic was higher in the older group (16–17 years), in those who belonged to any ethnic groups, and in children with a family member with asthma.

The prevalence of diagnosis bronchial asthma was 7.4%, and was significantly higher in males, in the age category between 13 and 15 years, in the ethnic group, and in children with a family member with asthma (Table 2). Regarding the sector of residence where the exposure occurred, the prevalence of bronchial asthma in those who lived in the sector where the stockpiles were accumulated (sector F) was higher than the prevalence in individuals who lived in the unexposed sector; however, no significant differences were observed.

The association models between the concentration of inorganic arsenic and bronchial asthma are presented in Table 3. The association was not significant when urinary arsenic was modeled as a continuous variable; however, when we categorized urine concentration into tertiles, children with concentrations ≥21.4 μg/L had a 1.90 times greater chance of developing asthma (95% CI 1.13–3.18) compared with children belonging to T1 after adjusting for age, sex, ethnicity, and urinary creatinine concentration.

We assessed whether ethnicity modified the effect of arsenic exposure on bronchial asthma. When stratified into three sub-groups of different ethnicity (not belonging, undefined, and belonging), the effect of arsenic exposure on asthma diagnosis increased in the subset of children belonging to any ethnic group (Table 4).

## 4. Discussion

This is the first study in Chile and Latin America to evaluate the association between arsenic exposure and diagnosis of bronchial asthma. Our results, based on a population of 1892 children and adolescents between 8 and 17 years, indicated that the concentration of inorganic arsenic in urine is, indeed, associated with the diagnosis of bronchial asthma. Based on official records of diseases, the estimated prevalence of bronchial asthma was 7.4%, lower than that estimated in a study of Chilean school children between 13 and 14 years (14.9%) [34]; however, it should be noted that our estimate was based on confirmed medical diagnosis, unlike the previous survey, which used the asthma questionnaire [3].

Consistent with the results of the present study, a recent cohort study of children born in Taiwan reported an overall prevalence of asthma of 8.4% at 8, 11, and 14 years of age. More importantly, that study demonstrated that pre-natal arsenic concentration was associated with asthma in children (OR 2.03; 95%CI [1.26–3.26]) [15]. A study in Bangladesh has also found a greater association of asthma-like symptoms in adults exposed to high concentrations of arsenic than individuals exposed to low concentrations, considering different exposure measures (water, hair, and nails) [17]. They also reported that intermediate (5.32–134 μg/L) and high (135–1800 μg/L) exposure levels of arsenic in water were associated with the level of serum IgE (β = 0.127; 95%CI [0.023–0.231] and β = 0.237; 95%CI [0.109–0.36], respectively). Similar results were obtained when evaluating arsenic exposure in hair and nails [18]. The authors also reported that type 2 cytokines increased with a higher concentration of arsenic in drinking water, hair, and nails [18]. Results in the same direction have been found with respect to serum periostin, which was positively associated with asthma symptoms and Th2 mediators [35].

Evidence regarding the effect of arsenic on the immune system provides an avenue to explain possible mechanisms of action on the development of childhood asthma. Several studies have shown that arsenic has an immunomodulatory effect on the synthesis of immunoglobulin E, Th2 cytokines (interleukins IL-4, IL-5, IL-13, and eotaxin), and periostin [17,18,35,36,37]. These mediators trigger the inflammatory reaction in airway cells, leading to the release of histamine, prostaglandins, and leukotrienes that produce asthma symptoms in children [38]. In addition, it has been documented that arsenic promotes the production of reactive oxygen species (ROS) [39] involved in the inflammatory response and the pathogenesis leading to the development of asthma [40].

Arsenic exposure in northern Chile represents a public health concern, as drinking water is the main source of exposure [41,42]. However, in the city of Arica, the concern regarding arsenic exposure was aggravated by the deposition and abandonment of 20,000 tons of toxic waste in a sector where social housing was built years later [21], this population being homogeneous in terms of low socio-economic level.

People belonging to some ethnic groups have additional vulnerability factors causing differential aspects in health [43]. Hence, we explored the joint effect of ethnicity and arsenic exposure on asthma diagnosis by stratified analysis, which indicated a marked effect of arsenic exposure (≥21.4 μg/L) on asthma among children belonging to any ethnic group (OR 3.51, 95%CI [1.43–8.65]).

To the best of our knowledge, no previous studies have analyzed the effect of arsenic and the development of childhood asthma by ethnicity. Instead, ample evidence points to racial and ethnic disparities in asthma prevalence [44]; even the observed racial differences in pediatric asthma have been reported to be maintained while controlling for socio-economic income and educational attainment [45]. Likewise, it has been described that exposure to heavy metals is typically greater in racial or ethnic minorities [46,47]; therefore, the results of this analysis point to the hypothesis that ethnic disparities disproportionately affect environmental hazards, due to their social condition.

Our study has limitations related to the cross-sectional nature of the data, which did not allow us to establish a causal relationship.

A second limitation is the use of data from a surveillance system, which limited us from having access to complete data (27% undefined ethnicity) or access on potential confounders such as socio-economic level or education, reducing the validity of the results obtained to some extent. However, in relation to this last aspect, as described above, this was a homogeneous population in terms of socio-economic level.

The large sample of subjects with measurement of urine inorganic arsenic was our main strength, benefiting from an accurate and adequate measure for the determination of exposure. In addition, we have complete urinary creatinine data, allowing us to adjust urinary inorganic arsenic values for variations in hydration or other factors that may influence concentration. Furthermore, the use of information from SIGGES increased the possibility that those who were considered asthmatic had been evaluated and diagnosed by health professionals, which allows us to assume the high validity of this measurement.

## 5. Conclusions

In this study, arsenic exposure was associated with bronchial asthma in children and adolescents, with the effect of arsenic exposure being greater among those belonging to any ethnic group. The results are consistent with the previous literature, demonstrating that arsenic affects airway inflammation, thus leading to the development of asthma. The results presented herein should be interpreted with caution, given the study’s limitations, which does not preclude consideration of public health measures aimed at reducing arsenic exposure to limit its impact on the respiratory health of children and adolescents.

## Figures and Tables

**Table 1:** Sociodemographic characteristics of the sample from Arica (2009–2021).

Characteristics	*n* [%]
Total	1892
Sex	
Female	958 [50.6]
Male	934 [49.4]
Age [years]	
8–12	697 [36.8]
13–15	717 [37.9]
16–17	478 [25.3]
Ethnicity	
Does not belong	788 [41.6]
Undefined	516 [27.3]
Belongs to any ethnic group	588 [31.1]
Sector of residence	
Outside the exposed sector	32 [1.7]
Other exposure sector	60 [3.2]
Sector F [exposed]	1800 [95.1]
Family asthma report ^1^	
No	1386 [92.2]
Yes	117 [7.8]

^1^*n* = 1503.

**Table 2:** Urinary arsenic concentration and prevalence of asthma according to individual characteristics, Arica (2009–2021).

	Urinary Arsenic μg/L		Prevalence of Asthma	
Characteristics	Median [IQR]	*p*-Value *	*n* [%]	*p*-Value **
Sex		0.233		0.037
Female	15 [7,8,9,10,11,12,13,14,15,16,17,18,19,20,21,22,23,24,25,26]		59 [6.1]	
Male	16 [8,9,10,11,12,13,14,15,16,17,18,19,20,21,22,23,24,25,26]		81 [8.7]	
Age [years]		<0.001		0.043
8–12	10 [5,6,7,8,9,10,11,12,13,14,15,16,17,18,19,20]		40 [5.7]	
13–15	18 [9,10,11,12,13,14,15,16,17,18,19,20,21,22,23,24,25,26,27,28]		66 [9.2]	
16–17	20 [11,12,13,14,15,16,17,18,19,20,21,22,23,24,25,26,27,28,29,30]		34 [7.1]	
Ethnicity		0.001		≤0.047
Does not belong	15 [8,9,10,11,12,13,14,15,16,17,18,19,20,21,22,23,24,25]		54 [6.8]	
Undefined	14 [6,7,8,9,10,11,12,13,14,15,16,17,18,19,20,21,22,23,24]		30 [5.8]	
Belongs to any ethnic group	17 [9,10,11,12,13,14,15,16,17,18,19,20,21,22,23,24,25,26,27,28]		56 [9.2]	
Sector of residence		0.509		0.646
Outside the exposed sector	14 [8,9,10,11,12,13,14,15,16,17,18,19,20,21,22]		1 [3.1]	
Other exposure sector	13 [8,9,10,11,12,13,14,15,16,17,18,19,20,21,22]		3 [5.0]	
Sector F [exposed]	15 [7,8,9,10,11,12,13,14,15,16,17,18,19,20,21,22,23,24,25,26]		136 [7.6]	
Family asthma report				
No	15 [7,8,9,10,11,12,13,14,15,16,17,18,19,20,21,22,23,24,25]	0.037	91 [6.6]	≤0.001
Yes	19 [8,9,10,11,12,13,14,15,16,17,18,19,20,21,22,23,24,25,26,27,28,29,30]		20 [17.1]	

* Wilcoxon rank-sum test (Mann–Whitney) for dichotomous variables and Kruskal–Wallis test for categorical variables. ** Pearson Chi-square test or Fisher’s exact test when frequencies < 5.

**Table 3:** Association between asthma and urinary arsenic concentration, Arica (2009–2021).

	Model 1 (*n* = 1892)		Model 2 (*n* = 1886)	
	OR	[95% CI]	OR	[95% CI]
Arsenic tertiles (T) μg/L				
T1 ≤ 10	Ref		Ref	
T2 10.1–21.3	1.46	[0.93, 2.28]	1.50	[0.93, 2.40]
T3 ≥ 21.4	1.71	[1.11, 2.62]	1.90	[1.13, 3.18]

Model 1: Simple association model between arsenic and bronchial asthma diagnosis. Model 2: Adjusted for age (years), sex (female: 0, male: 1), ethnicity (not belonging: 1, undefined: 2, belonging: 3), and creatinine (g/L).

**Table 4:** Modifying effect of ethnicity on the association between urinary arsenic concentration and bronchial asthma in children, Arica (2009–2021).

	Model 1 (*n* = 786)		Model 2 (*n* = 513)		Model 3 (*n* = 587)	
Ethnicity	Not Belonging		Undefined		Belonging	
	OR	[95% CI]	OR	[95% CI]	OR	[95% CI]
Arsenic tertiles (T) μg/L						
T1 ≤ 10	Ref		Ref		Ref	
T2 10.1–21.3	1.41	[0.69, 2.89]	0.79	[0.26, 2.40]	2.47	[1.05, 5.79]
T3 ≥ 21.4	1.06	[0.46, 2.45]	2.36	[0.79, 7.00]	3.51	[1.43, 8.65]

Models 1, 2, and 3 adjusted for age (years), sex (female: 0, male: 1), and creatinine (g/L).
